# Using the Y-balance Test as a Predictor Tool for Evaluating Non-contact Injuries in University League Football Players: A Prospective Longitudinal Study

**DOI:** 10.7759/cureus.39317

**Published:** 2023-05-21

**Authors:** Khalid M Alkhathami

**Affiliations:** 1 Health Rehabilitation, Shaqra University, Shaqra, SAU

**Keywords:** cutoff scores, predicting injury, clinical prediction rules, injury incidence, soccer, dynamic balance

## Abstract

Background

Football is a highly competitive sport, and participants can experience various contact and non-contact sports injuries in the sporting process. In any elite sport, screening players using different scientific tools is an important injury prevention strategy. The Y- Balance test (YBT) was found to be a predictive tool for non-contact injury. However, the use of criteria from these tests to predict injuries has not been substantiated and should be further investigated.

Purpose

The aim of this study was to determine the predictors for injury among athletes using baseline YBT, number of matches, and minutes of physical activity; the cutoff scores for predictors of injury, including baseline YBT, number of matches, and minutes of physical activity; and the clinical prediction rules for predicting injury in this population.

Methods

A total of 39 young student football players were included in this study. The mean age was 20.28 years, and the mean body mass index (BMI) was 23.83 kg/m^2^. A baseline assessment of the participant’s characteristics was taken and each participant performed the YBT once before starting the league. After the university league football players had finished their tournament, we asked them questions related to non-contact injuries.

Results

The results showed that the prevalence of injury was 17.95% among this population. An increase in the YBT score was significantly associated with a decrease in the odds of having an injury [odds ratio (OR) 95% confidence interval (CI): 0.94 (0.88, 0.99), *p* = 0.047). In addition, the number of matches was significantly associated with an increase in the odds of having an injury *p* = 0.012. However, the minutes of physical activity were not statistically significant *p* = 0.065. The highest Youden index was ≤97.89, with a sensitivity of 87.50% and specificity of 71.43%, for the posterior medial reach and ≤92.88, with a sensitivity of 90.62% and specificity of 57.14%, for the posterior lateral reach. The clinical prediction rule was an area under the curve (AUC) of 0.88.

Conclusions

The results of the study provide evidence for the potential utility of the YBT as a predictor tool for evaluating non-contact injuries in university league football players. By identifying players with lower YBT scores who were at higher risk for injury, targeted interventions could be implemented to address functional movement deficits and potentially reduce injury risk.

## Introduction

Football (soccer) is, indisputably, the most popular team sport in the world, and more than 220 million players worldwide are currently estimated to participate in organized football [1]. Football is the most popular national sport in Saudi Arabia. Playing football as a leisure activity is very common [2]. Football has potential positive effects on players’ metabolic, cardiovascular, and musculoskeletal parameters, alongside a substantial risk of contact and non-contact injury [3,4]. Football injuries can result in both financial and time-related costs. In an elite professional team playing football, prevention of injury is paramount in protecting the playing form of the players. Limiting the time lost through injury is important to maintain the performance of the team and safeguard the financial interests involved in the game [5]. One study on football reported increases in subsequent injury risk for every previous injury sustained, highlighting the dangers of falling into a cycle of repeated injuries and the associated consequences, such as poor performance, deselection, or retirement [6]. Given that non-time loss injuries are associated with an elevated subsequent injury risk [7], identification of unipedal postural balance asymmetry in soccer players may highlight their risk of sustaining subsequent noncontact lower extremity injuries. Subsequent injuries tend to be more severe than the initial ones owing to their greater sports incapacity (time-loss from sport) and larger burden on sports medical resources [8]. Thus, there is an urgent need to screen subsequent noncontact lower extremity musculoskeletal injuries occurrence in soccer players. Such data could help sports coaches and clinicians make return-to-play decisions and provide them with additional information when designing training and rehabilitation programs. In any elite sport, screening players using different scientific tools comprises an important part of injury prevention [8,9].

One scientific screening tool is the Y-Balance Test (YBT), which was developed as a standardized version of the modified Star Excursion Balance Test (SEBT) [10]. It can efficiently detect deficiencies in a player’s trunk stability, balance, and neuromuscular control to assess a person’s risk for injury [11]. It is used to assess dynamic balance impairments associated with lower extremity injuries [12]. In football, the unipedal postural balance is a fundamental component of performance [13]. Its deterioration is associated with performance decrease and injuries [14]. It is a simple and reliable method of assessing the subject’s stability, balance, and strength in various directions. Several studies have shown the YBT to have excellent test-retest reliability between raters and a minimal measurement error [15,16]. During the YBT, the subject is required to balance on one leg and reach in three directions, anterior, posteromedial, and posterolateral, as far as they can. The composite score of the YBT is computed by adding the reach in all three directions and then normalizing the result according to the length of the lower limb. Asymmetry is calculated as the difference between the reach of the right and left limbs [17].

Butler et al. [17] found that the composite YBT score is predictive of non-contact lower extremity injury, but there was no association between injury and asymmetry in any of the reach directions in collegiate American football players. A normalized composite score below 89.6% in the YBT is associated with an increased risk of non-contact lower extremity injury [17]. In addition, high school female athletes with YBT composite scores below 94% were 6.5 times more likely to experience a lower extremity injury [18]. YBT composite scores vary across sports and levels of competition [18-21]. Previous research suggests that soccer players score higher on dynamic balance compared to basketball players when measured by normalized leg reach distances on the SEBT [21]. The literature also indicates that gymnasts and dancers have superior balance compared to soccer players, as assessed through the center of pressure sway index [22,23]. Performance on the YBT test varies depending on the competitive level, sport, gender, and age [18-21,24,25]. Therefore, the use of criteria from these tests to predict injuries has not been substantiated and should be further investigated. Moreover, the sensitivity and specificity of the YBT as a tool for injury prediction in football have not yet been reported, and there have been no studies investigating the clinical prediction rules with football players.

Determining normative scores specific to a population may help in identifying injury-risk thresholds and return-to-play criteria following an injury. Therefore, this study had three main objectives: (1) to examine the predictors for injury among athletes using baseline YBT, number of matches, and minutes of physical activity; (2) to identify the cutoff scores for predictors of injury, including baseline YBT, number of matches, and minutes of physical activity; and (3) to establish clinical prediction rules for predicting injury in this population.

## Materials and methods

Study design

This study was a longitudinal prospective study from October 2022 to December 2022. It was carried out at the Department of Rehabilitation Sciences in the College of Applied Medical Sciences, Shaqra University. This study followed the Declaration of Helsinki guidelines for principles of human experimentation. Ethical approval was obtained from the Ethical Research Committee at Shaqra University (ERC_SU_20210068) before data collection.

Participants

In this study, healthy athlete subjects were recruited. The sample in this study was 39 young players who were students at Shaqra University. A convenience sample of participants was recruited for the study through the Shaqra University email system, local advertising, and word of mouth. The eligible participants were male football players aged 18-25 years, who were free from musculoskeletal injuries at the time of testing. Participants were excluded from the study if they were receiving drugs for medical conditions or had any cardiovascular, metabolic, respiratory, orthopedic, or neurological conditions contraindicating the test.

 Instrumentation

**Figure 1:**
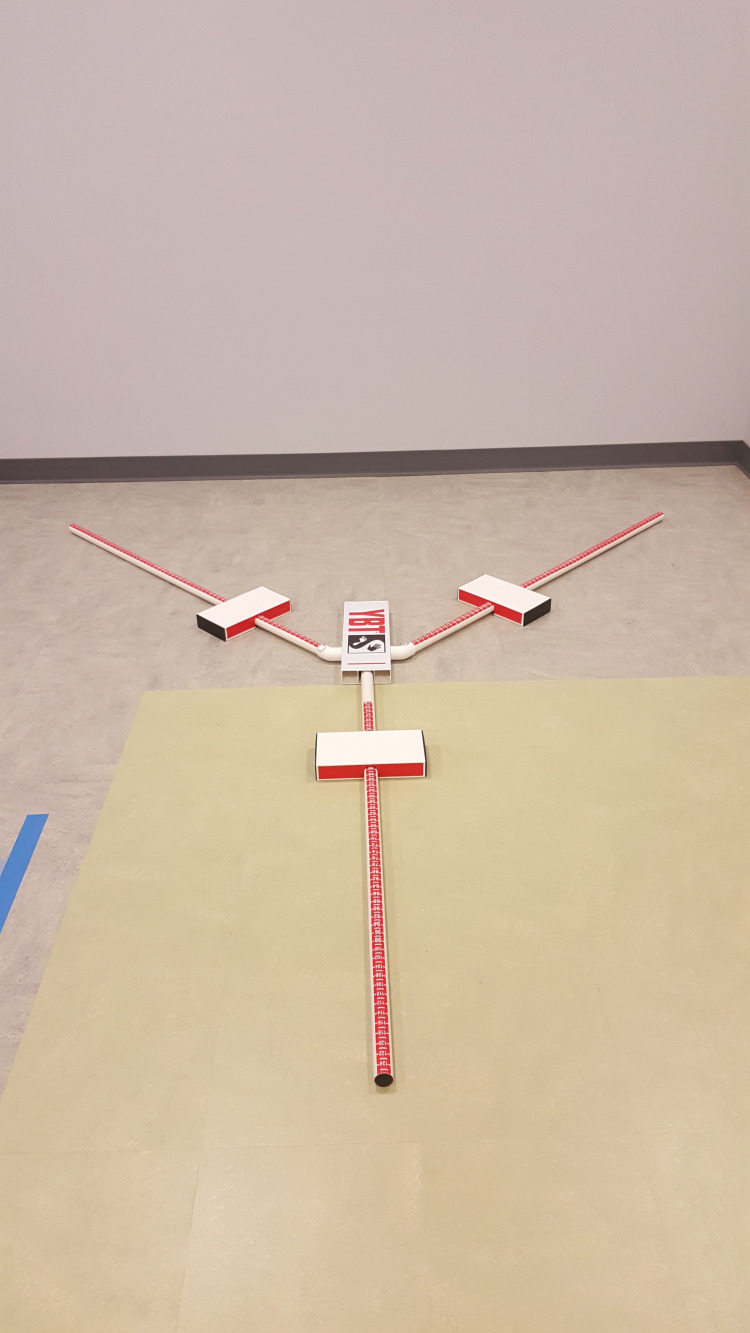
Y-Balance Test Kit

The Y-Balance Test™ Kit (functionalmovement.com, Danville, VA, USA) was used at baseline in this study, which is a device comprised of a central plastic plate, to which three tubes are attached in three directions: the anterior, posteromedial, and posterolateral reach directions Figure 1.

The reaching direction is named in orientation to the stance limb [17]. For measurement, each pipe is marked in five-millimeter increments. The YBT is a reliable measure that was developed from the star excursion balance test [11]. Intra-tester reliability estimates [intraclass correlation coefficients (ICC)] for the YBT device ranged from 0.87 to 0.88, and the inter-tester reliability ranged from 0.88 to 0.90 [26]. The reach distances were normalized to each participant’s leg length as a percentage of this measurement. Normalization was performed by dividing each excursion distance by the participant’s leg length and then multiplying by hundreds. Normalized values were a percentage of the excursion distance relative to a participant’s leg length [16].

Procedures

Before the data collection began, eligible and interested participants were invited to attend the College of Applied Medical Sciences for a single testing session in the therapeutic exercise lab. The session lasted for a half hour, and the subjects wore their own sportswear (shorts and t-shirt). Before starting the YBT assessment, participants were provided with basic information about using the YBT instrument and its tests.

At the session, they were provided information about the study, and their rights as human participants, and were asked to sign the informed consent form before partaking in the study. The eligible and consenting participants were allocated for the test. Before the measurement, a baseline assessment of the participant’s characteristics was taken by the investigator, followed by the YBT. Upper-limb dominance was determined by asking the subject to write their name and sign the consent form, while lower-limb dominance was determined by asking the volunteer to kick a ball three times. The YBT assessments were measured in the morning between (8:00 a.m. to 11:00 a.m.). The body weight and height were used to calculate the body mass index. The leg length was measured bilaterally in centimeters and was used to normalize reach distances because leg length has been shown to be a factor affecting YBT performance [27].

All participants were shoeless when performing the YBT measurements. Each participant was asked to slide a reach indicator along a pipe in the three testing directions: anterior, posteromedial, and posterolateral on both legs. The procedure for measurement consisted of nine attempts (citation). The first six attempts were considered trials in order to familiarize the participant with the test procedure. The YBT was conducted by a qualified, experienced physical therapist who was responsible for giving verbal instructions and visual demonstrations of the YBT, as well as recording the YBT measures. The trial was discarded and retried when the participant (1) moved the foot of the stance leg from the platform or crossed the marked line, (2) kicked, pushed, or stepped on the reach indicator, (3) touched the floor with the reaching leg, or (4) lost balance before returning to the standing position. To reduce fatigue, participants were given a rest of a minimum of 10 s between each reach and 30 s between each direction of testing. The reach distance was measured to the nearest centimeters by reading the line at the proximal edge of the reach indicator. Three successful test trials in each direction on each leg were recorded and normalized to the leg length, and the composite score for each direction and all directions were used for data analysis [16].

After the university league football players finished their tournament, we contacted them and asked the following questions related to the evaluation of non-contact injuries: How many matches have you played? How long was the average game time you played? Do you do stretches and warm-up before the match? How many minutes per week do you do physical activity? Did you have muscle spasms or fatigue after the match? Did you get injured during a match or training? Injury location and description were documented.

Statistical analysis

The incidence of injury was considered a binary categorical variable. The incidence of injury was dichotomized as yes (have an injury) or no (no injury). Descriptive statistics for demographics and clinical variables were expressed as means with standard deviations (SD) for continuous variables and counts (n) with percentages for categorical variables. Comparisons between athletes with and without injuries were made using an independent t-test for continuous variables.

To examine the association between scores of the YBT and the incidence of injury among university football players during their competition period, multivariable binary logistic regression was used with odds ratios (OR) and 95% confidence intervals (95% CI). The model was adjusted for age, sex, and body mass index (BMI). The reference category for the main outcome (dependent variable) was set as no injury.

To determine the optimal cutoff score for YBT that was associated with the incidence of injury among university football players during their competition period, the receiver operating characteristic (ROC) curve was utilized with an area under the curve (AUC) to show the overall accuracy of the model in distinguishing players with injury from players without injury at specified cutoff scores. The Youden index was used to determine the optimal cutoff score for the YBT using this formula: Youden index = [sensitivity + (1 − specificity)]. The sensitivity and specificity were calculated to estimate true positive and true negative results, respectively. Finally, clinical prediction rules were calculated using significant associated risk factors, including the number of matches, physical activities, and the YBT at specified cutoff scores for each variable. Each variable was categorized as yes or no based on the optimal cutoff score associated with the incidence of injury. Then, a unique variable was created by compounding mutually independent factors and tests to discriminate between participants with injury incidence and non-injury. To avoid multicollinearity, we included posterior medial reach and posterior lateral reach, and we excluded the YBT composite score. The sensitivity, specificity, positive likelihood ratio, and negative likelihood ratio were calculated for the clinical prediction rules. An alpha level of 0.05 was used for analysis. We used IBM SPSS for Mac version 25.0 (SPSS Inc. Chicago, IL, USA) and STATA for Mac version 14.1 (Stata Corp, College Station, TX, USA) for all data analyses.

## Results

A total of 39 young student football players were included in this study. The prevalence of injury was 17.95% in this population. The mean age was 20.28 years, and the mean BMI was 23.83 kg/m2. Table 1 shows the demographics, clinical factors, and YBT scores of those with injury and those without. The number of matches played, posterior medial reach, posterior lateral reach, and the YBT composite score significantly differed between the two groups.

**Table 1:** Participant demographics and clinical factors. * p-value was based on independent t-test for continuous variables, SD: standard deviation, BMI: body mass index, Ant Reach: anterior reach, Post Med Reach: posteromedial reach, Post Lat Reach: posterolateral reach. YBT: Y- balance test.

Factors	Non-Injury (n = 32)	Injury (n = 7)	p-Value *
Age, years (mean ± SD)	20.31 ± 1.35	20.14 ± 1.57	0.772
BMI, kg/m^2^ (mean ± SD)	23.80 ± 2.78	23.96 ± 3.22	0.890
Number of matches played (mean ± SD)	1.63 ± 1.04	3 ± 1.30	0.004
Average game time played (mean ± SD)	26.56 ± 16.82	37.86 ± 16.80	0.116
Physical activity minutes per week (mean ± SD)	167 ± 95.93	95.71 ± 34.57	0.062
Ant Reach (mean ± SD)	80.21 ± 16.40	71.64 ± 12.44	0.202
Post Med Reach (mean ± SD)	112.78 ± 18.72	93.18 ± 15.15	0.016
Post Lat Reach (mean ± SD)	112.02 ± 19.10	94.53 ± 15.67	0.030
YBT Composite score (centimeter) (mean ± SD)	101.53 ± 16.51	86.58 ± 13.26	0.032

**Figure 2:**
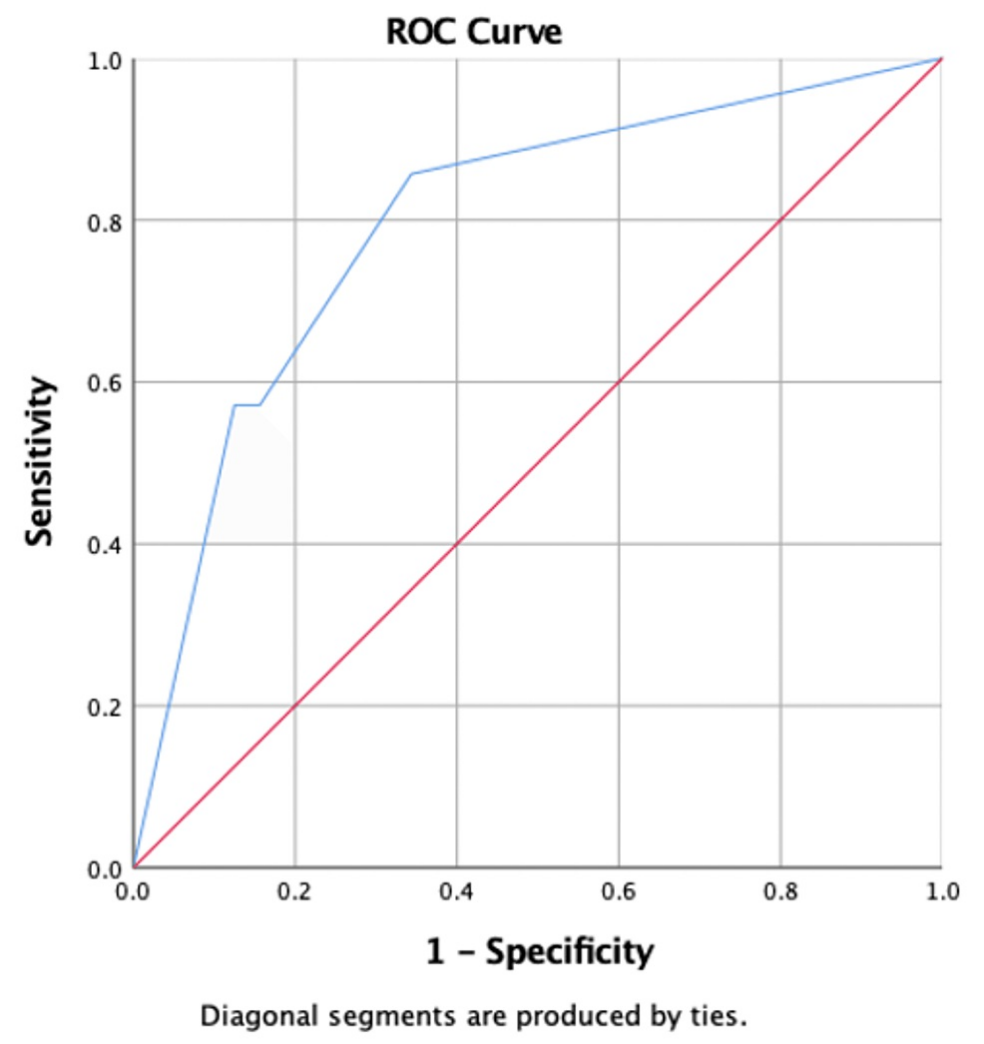
Receiver Operating Characteristics (ROC) curve and cut-off score for the number of matches played associated with the incidence of injury.

**Figure 3:**
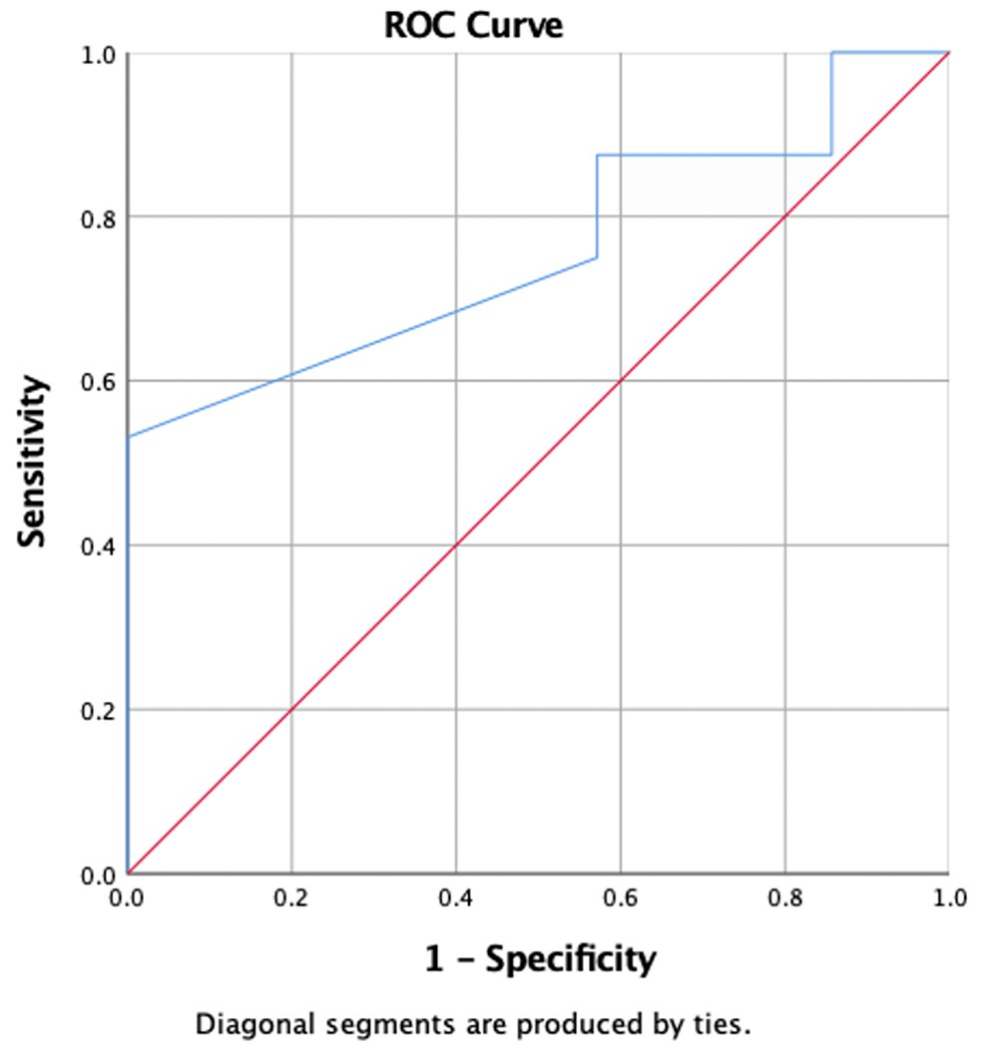
Receiver Operating Characteristics (ROC) curve and cut-off score for physical activity associated with the incidence of injury.

The results of the multivariable logistic regression examining the association between anterior reach, posterior medial reach, posterior lateral reach, and the YBT composite score and injury are shown in Table 2. An increase in the YBT score was significantly associated with a decrease in the odds of having an injury (OR 95% CI: 0.94 (0.88, 0.99), p = 0.047). This indicates that with every increase in the YBT score, there was a decrease of 6% in the incidence of injury. In addition, the number of matches was significantly associated with an increase in the odds of having an injury, p = 0.012 Table 2 and Figure 2. However, the minutes of physical activity were not statistically significant p = 0.065 Table 2 and Figure 3.

**Table 2:** Binary logistic regression for injury versus YBT score, number of matches, and minutes of physical activity. YBT: Y- balance test, OR: odds ratio, CI: confidence interval, * after controlling for age and BMI.

Factor	OR (95% CI)	p-Value
Total YBT score *	0.94 (0.88, 0.99)	0.047
The number of matches *	2.78 (1.26,6.17)	0.012
Minutes of physical activity*	0.98 (0.96, 1.00)	0.065

**Figure 4:**
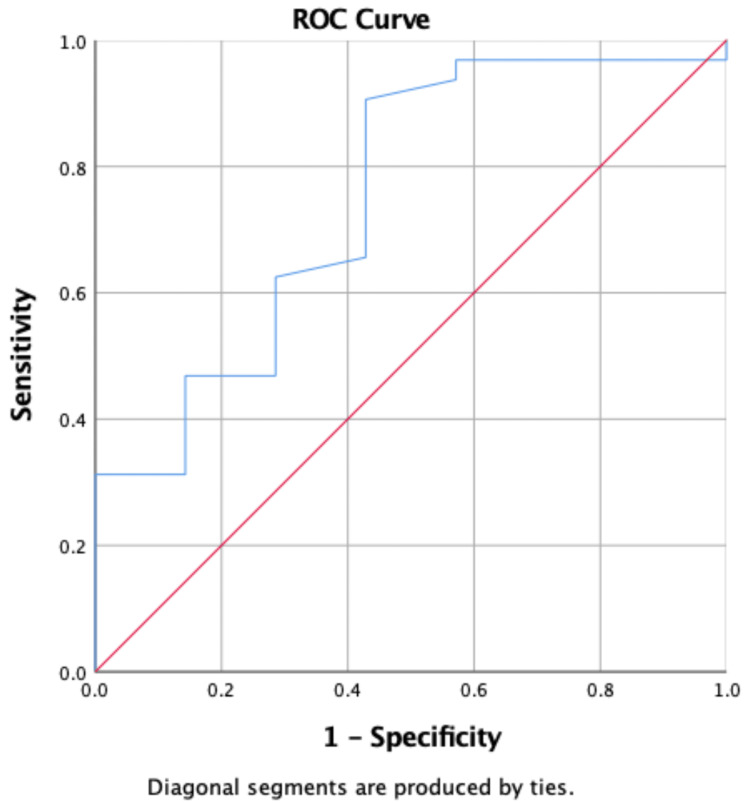
Receiver Operating Characteristics (ROC) curve and cut-off score for the posterior lateral reach score associated with the incidence of injury.

**Figure 5:**
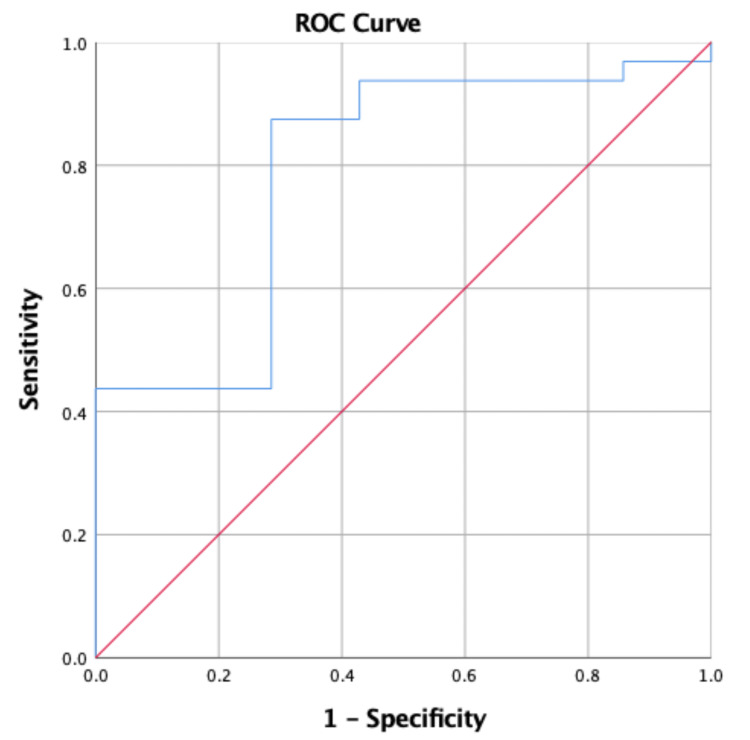
Receiver Operating Characteristics (ROC) curve and cut-off score for the posterior medial reach score associated with the incidence of injury.

The results of the receiver operator characteristics examining the optimal cutoff of the YBT score associated with injury are shown in Table 3 and Figures 4, 5. The results showed that the optimal cutoff with the highest Youden index was ≤97.89, with a sensitivity of 87.50% and specificity of 71.43%, for the posterior medial reach and ≤92.88, with a sensitivity of 90.62% and specificity of 57.14%, for the posterior lateral reach. The area under the curve was 0.79 for posterior medial reach and 0.75 for posterior lateral reach, indicating fair accuracy.

**Table 3:** Receiver Operating Characteristics (ROC) curve and cut-off score for number of matches played, physical activity, posterior medial reach score, and posterior lateral reach score associated with incidence of injury. AUC: area under the curve, * selected by highest Youden index.

Variables	AUC (95% CI)	Cut-Off Score (Sensitivity, Specificity) *
Number of matches played	0.80 (0.61, 0.98)	≥2 (85.71%, 65.62%)
Physical Activity	0.76 (0.60, 0.92)	≤90 (87.50%, 42.86%)
Posterior Medial Reach	0.79 (0.60, 0.98)	≤97.89 (87.50%, 71.43%)
Posterior Lateral Reach	0.75 (0.54, 0.96)	≤92.88 (90.62%, 57.14%)

**Figure 6:**
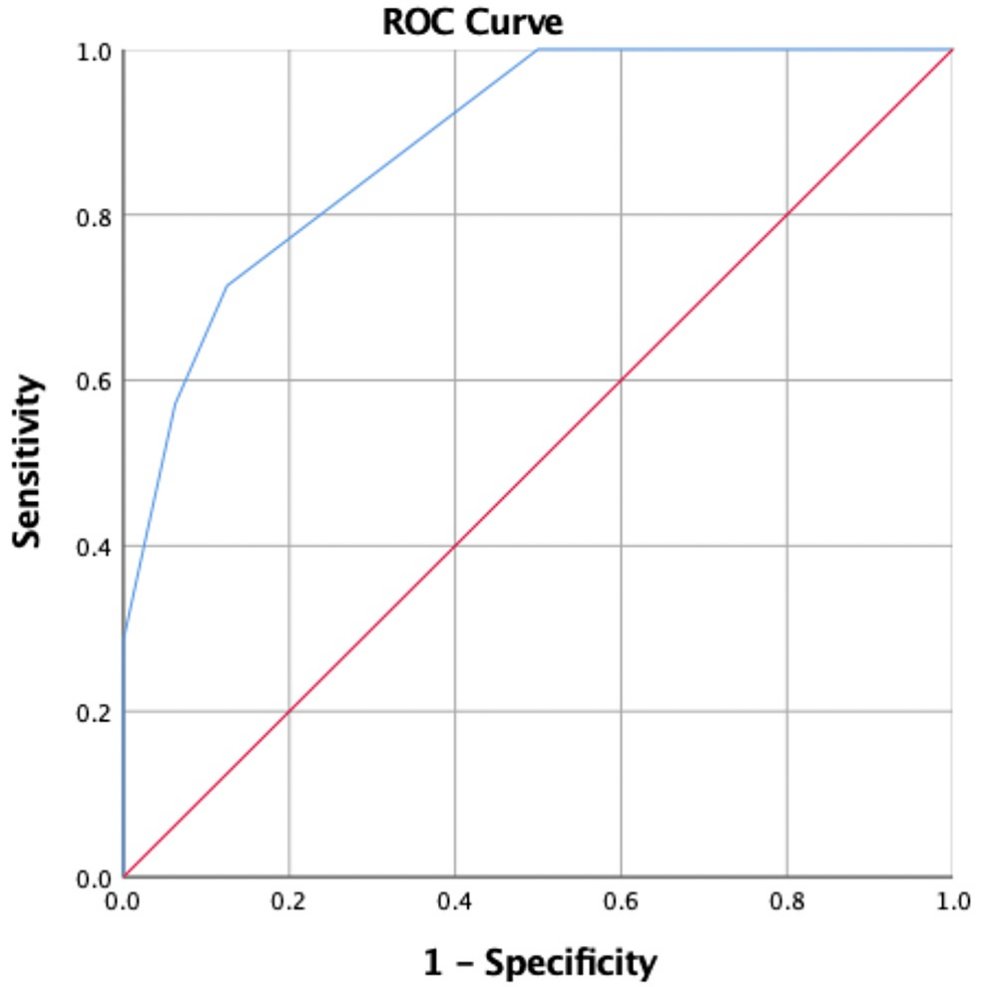
Receiver Operating Characteristics (ROC) curve and cut-off score for the clinical prediction rules for predicting injury.

The results for the clinical prediction rules showed that posterior medial reach of YBT scores, posterior lateral reach of YBT scores, number of matches played, and minutes of physical activity might provide clinical prediction rules, AUC (95% CI) 0.88 (0.76%, 0.97%), with a cut-off score of ≥3 (sensitivity 57.14%, specificity 93.75%) Table 4 and Figure 6.

**Table 4:** Receiver Operating Characteristics (ROC) curve and cut-off score for the clinical prediction rules for predicting injury. AUC: area under the curve. * selected by highest Youden index.

Variables	AUC (95% CI)	Cut-off Score (Sensitivity, Specificity) *
Clinical prediction rules	0.88 (0.76%, 0.97%)	≥3 (57.14%, 93.75%)

## Discussion

In this study, we aimed to investigate the YBT as a predictor tool for evaluating non-contact injuries in university league football players who had no lower extremity injuries or problems before starting the soccer university league. The longitudinal design of the study allowed us to monitor the players throughout an entire season, providing valuable insights into injury patterns and the potential benefits of using YBT as an assessment tool. The findings from this study demonstrated a significant association between YBT performance and the occurrence of non-contact injuries in university league football players. There was a 6% decrease in the odds of incurring an injury with every one-point increase in the YBT composite score [odds ratio (OR): 0.94, 95% confidence interval (CI): 0.88 to 0.99, p = 0.047)]. This indicates that players with better dynamic balance may have a lower risk of lower extremity injuries. Players with lower YBT scores were more likely to sustain non-contact injuries during the season, highlighting the potential utility of the YBT as a predictor of injury risk. The findings of this study support previous research suggesting that functional movement deficits, as assessed by the YBT, may be linked to a higher likelihood of injury [10,17].

The YBT test was able to identify the risk of injuries. Some studies mentioned both specificity (true negative rate) and sensitivity (true positive rate). The sensitivity is a more important metric than the specificity metric [28]. In addition, the YBT test sensitivity is important to rule out potential injury [29]. We extended the prior research by identifying optimal YBT cutoff scores associated with injury risk. Players with a posteromedial reach score of less than 97.89 cm and a posterolateral reach score of less than 92.88 cm were at higher risk of sustaining a lower extremity injury. These cutoff scores can potentially be used by clinicians and coaches to identify at-risk players who may benefit most from balance training and tailored injury prevention programs. The receiver operating characteristic analyses also demonstrated fair accuracy for these selected reach directions (AUC: 0.75 to 0.79), indicating that the YBT has potential value as a screening tool to predict injury risk. These findings were consistent with those of previous studies that have identified YBT as a valid and reliable screening tool for assessing lower extremity injury risk [10,17,18]. However, Wright et al. reported that the YBT did not predict general lower extremity injury in Division I athletes from multiple sports. These researchers suggested that the YBT has been employed to describe the movement and balance capabilities of athletes [30]. However, they argued the use of criteria from these tests to predict injuries had not been substantiated and should be further investigated. A possible explanation for this discrepancy could be differences in the populations studied or variations in study methodology. For example, methodological limitations in some of the included studies, such as small sample sizes, lack of multivariate analyses, and inconsistent reporting of balance test scores, may have also contributed to the inconclusive findings. In contrast, the association between injury incidence and the number of matches played and minutes of physical activity was less consistent. While there was a significant association between the number of matches played and injury risk p = 0.012, the association between minutes of physical activity and injury risk was not statistically significant p = 0.065. This discrepancy may be due to differences in the intensity and frequency of physical activity during matches compared to other types of physical activity, as well as the potential influence of other external factors, such as match conditions, player position, and level of competition. The clinical prediction rules demonstrated a high level of accuracy for predicting injury risk, with an AUC of 0.88 and a selected cutoff score of ≥3 (sensitivity: 57.14%, specificity: 93.75%). This suggests that the combination of YBT scores, number of matches played, and minutes of physical activity may provide a more comprehensive assessment of injury risk compared to individual variables alone. However, further research is needed to validate and refine these clinical prediction rules in larger and more diverse populations of athletes.

The findings of this study carry important implications for injury prevention strategies in university league football players. By identifying players with lower YBT scores who are at higher risk for non-contact injuries, coaches and athletic trainers could implement targeted interventions to address functional movement deficits and potentially reduce injury risk. These interventions may include individualized exercise programs, neuromuscular training, and balance training, which have been shown to improve YBT performance and reduce injury risk in previous research [31-33]. Future research should continue to explore the relationships between YBT scores, other injury risk factors, and sport-specific performance measures to optimize injury prevention strategies and enhance our understanding of the underlying mechanisms contributing to injury risk. Furthermore, our results indicated that the YBT may be a valuable tool for monitoring functional movement abilities in football players throughout the season. Regular assessments may enable coaching staff to track changes in YBT performance and identify players who may be at an increased risk of injury due to declines in functional movement abilities. Finally, the observation that injury risk may be influenced by playing position highlights the need for position-specific injury prevention strategies in football. Future research should investigate the role of the YBT in predicting injury risk for different playing positions, and the effectiveness of targeted interventions for addressing position-specific functional movement deficits.

This study was not without limitations. Firstly, we used a convenience sample of a single university league, which may limit the generalizability of our findings to other populations. Future research should examine the role of the YBT in predicting injury risk in other populations, such as professional football players, youth athletes, and female athletes. Secondly, our study focused solely on non-contact injuries. While this allowed us to examine the relationship between YBT performance and injury risk in a more homogeneous injury population, it is important to note that the majority of injuries in football are contact-related [33]. Future research should investigate the role of the YBT in predicting contact injuries and the potential benefits of using the YBT as part of a broader injury prevention strategy. Lastly, the observational nature of our study prevents us from making causal inferences about the relationship between YBT scores and injury risk. Future research should employ experimental designs to investigate the causal relationship between YBT performance and injury risk, and to determine the effectiveness of interventions aimed at improving YBT performance for reducing injury risk.

## Conclusions

Our study provides evidence for the potential utility of the YBT as a predictor tool for evaluating non-contact injuries in university league football players. Specifically, players with a lower posteromedial and posterolateral reach distance may benefit from targeted injury prevention programs to reduce their injury vulnerability. By identifying players with lower YBT scores who are at higher risk for injury and considering the number of matches played and minutes of physical activity, targeted interventions can be implemented to address functional movement deficits and potentially reduce injury risk. Future research should continue to explore the role of the YBT in predicting injury risk and the effectiveness of interventions for improving YBT performance in football players. With further research, the Y-Balance Test could be an efficient way for coaches and sports medicine staff to implement selective injury prevention strategies.
